# Prevalence of Germline Alterations on Targeted Tumor-Normal Sequencing of Esophagogastric Cancer

**DOI:** 10.1001/jamanetworkopen.2021.14753

**Published:** 2021-07-12

**Authors:** Geoffrey Y. Ku, Yelena Kemel, Steve B. Maron, Joanne F. Chou, Vignesh Ravichandran, Zarina Shameer, Anna Maio, Elizabeth S. Won, David P. Kelsen, David H. Ilson, Marinela Capanu, Vivian E. Strong, Daniela Molena, Smita Sihag, David R. Jones, Daniel G. Coit, Yaelle Tuvy, Kendall Cowie, David B. Solit, Nikolaus Schultz, Jaclyn F. Hechtman, Kenneth Offit, Vijai Joseph, Diana Mandelker, Yelena Y. Janjigian, Zsofia K. Stadler

**Affiliations:** 1 Department of Medicine, Memorial Sloan Kettering Cancer Center, New York, New York; 2 Department of Medicine, Weill Cornell Medical College, New York, New York; 3 Niehaus Center for Inherited Cancer Genomics, Memorial Sloan Kettering Cancer Center, New York, New York; 4 Department of Epidemiology & Biostatistics, Memorial Sloan Kettering Cancer Center, New York, New York; 5 Now with AstraZeneca, Gaithersburg, Maryland; 6 Department of Surgery, Memorial Sloan Kettering Cancer Center, New York, New York; 7 Department of Surgery, Weill Cornell Medical College, New York, New York; 8 Marie-Josée & Henry R. Kravis Center for Molecular Oncology, Memorial Sloan Kettering Cancer Center, New York, New York; 9 Human Oncology and Pathogenesis Program, Memorial Sloan Kettering Cancer Center, New York, New York; 10 Department of Pathology, Memorial Sloan Kettering Cancer Center, New York, New York

## Abstract

**Importance:**

Among patients with esophagogastric cancers, only individuals who present with known features of heritable cancer syndromes are referred for genetic testing. Broader testing might identify additional patients with germline alterations.

**Objectives:**

To examine the prevalence of likely pathogenic or pathogenic (LP/P) germline alterations among patients with esophagogastric cancer and to assess associations between germline variant prevalence and demographic and clinicopathologic features.

**Design, Setting, and Participants:**

This cross-sectional study was performed at a tertiary referral cancer center from January 1, 2014, to December 31, 2019, in 515 patients with esophagogastric cancer who consented to tumor and blood sequencing.

**Main Outcomes and Measures:**

Presence or absence of LP/P variants in up to 88 genes associated with cancer predisposition syndromes as identified by targeted sequencing (Memorial Sloan Kettering–Integrated Mutation Profiling of Actionable Cancer Targets).

**Results:**

Among 515 patients (median age, 59 years; range, 18-87 years; 368 [71.5%] male; 398 [77.3%] White), 243 (47.2%) had gastric cancer, 111 (21.6%) had gastroesophageal junction (GEJ) cancer, and 161 (31.3%) had esophageal cancer. A total of 48 patients with gastric cancer (19.8%), 16 (14.4%) with GEJ cancer, and 17 (10.6%) with esophageal cancer had LP/P germline variants. The number of LP/P variants in high- and moderate-penetrance genes was significantly higher in patients with gastric cancer (29 [11.9%]; 95% CI, 8.1%-16.7%) vs patients with esophageal cancer (8 [5.0%]; 95% CI, 2.2%-9.6%; *P* = .03), and the difference was greater for high-penetrance germline alterations in patients with gastric cancer (25 [10.3%]; 95% CI, 6.8%-14.8%) vs in patients with esophageal cancer (3 [1.9%]; 95% CI, 0.38%-5.3%; *P* = .001). The most frequent high- and moderate-penetrance LP/P alterations were in *BRCA1/2* (14 [2.7%]), *ATM* (11 [2.1%]), *CDH1* (6 [1.2%]), and *MSH2* (4 [0.8%]). Those with early-onset disease (≤50 years of age at diagnosis) were more likely to harbor an LP/P germline variant (29 [21.0%]; 95% CI, 14.5%-28.8%) vs those with late-onset disease (patients >50 years of age at diagnosis) (52 [13.8%]; 95% CI, 10.5%-17.7%; *P* = .046). *ATM* LP/P variants occurred in 6 patients (4.3%; 95% CI, 1.6%-9.1%) with early-onset esophagogastric cancer vs 5 (1.3%; 95% CI, 0.4%-3.1%; *P* = .08) of those with late-onset esophagogastric cancer.

**Conclusions and Relevance:**

These results suggest that pathogenic germline variants are enriched in gastric and early-onset esophagogastric cancer and that germline testing should be considered in these populations. The role of *ATM* alterations in esophagogastric cancer risk warrants further investigation.

## Key Points

**Question:**

What are the prevalences of likely pathogenic or pathogenic (LP/P) germline variants in esophageal, gastroesophageal junction, and gastric cancers?

**Findings:**

In this cross-sectional study of 515 patients, LP/P germline variants occurred in approximately one-fifth of patients with gastric cancer. Those with early-onset esophagogastic cancer (≤50 years of age at diagnosis) were significantly more likely to harbor an LP/P germline variant.

**Meaning:**

The results of this study support germline testing in patients with gastric cancer and early-onset esophagogastric cancer.

## Teaser

This genetic association study examines the prevalence of likely pathogenic or pathogenic germline alterations among patients with esophagogastric cancer and assesses associations between germline variant prevalence and demographic and clinicopathologic features.

## Introduction

Esophagogastric cancer is one of the most common cancers globally, with more than 1 million cases diagnosed annually.^1^ Although most esophagogastric cancers are sporadic,^2^ gastric cancer can arise within the context of heritable cancer predisposition syndromes, specifically hereditary diffuse gastric cancer, hereditary breast and ovarian cancer, and Lynch syndrome.^3^ However, the prevalence of pathogenic germline variants in patients with esophagogastric cancer remains poorly defined because only individuals who present with features of these heritable cancer syndromes are routinely referred for genetic testing.^4^

We retrospectively reviewed patients with esophagogastric cancer who underwent targeted sequencing of tumor tissue and blood (to provide a germline comparison) using Memorial Sloan Kettering–Integrated Mutation Profiling of Actionable Cancer Targets (MSK-IMPACT), a next-generation sequencing platform. We investigated whether the prevalence of likely pathogenic/pathogenic (LP/P) germline variants differed according to tumor location, age of diagnosis, and race/ethnicity and evaluated the association between the presence of germline variants and response to platinum-based chemotherapy regimens.

## Methods

### Patients

In this cross-sectional study, we identified all patients with esophagogastric cancer whose tumors and matched germline DNA from blood were analyzed by MSK-IMPACT at the Memorial Sloan Kettering Cancer Center (MSKCC) from January 1, 2014, to December 31, 2019; in general, testing was offered to patients who were followed up at MSKCC and who would potentially be candidates for an experimental strategy based on the identification of an actionable somatic alteration. Starting in May 2015, patients were prospectively offered secondary germline analysis after providing written informed consent for tumor genetic analysis in the context of an MSKCC institutional review board–approved protocol. The identities of those who provided consent only for somatic testing were anonymized before analysis.

Patients underwent anonymized or identified germline analysis, the latter in a prospective study starting in May 2015, with results returned to the patient. For the anonymized analysis, only patients who consented to the MSKCC institutional review board–approved prospective study of tumor genomic analysis via MSK-IMPACT were analyzed. A retrospective protocol for anonymized assessment of germline cancer susceptibility using normal DNA was approved by the MSKCC institutional review board. In this cohort, patient identities were anonymized after demographic data were summarized before other analyses. Institutional procedures for anonymization were followed to prevent inadvertent patient identification, with binning of clinical data into categorical variables of at least 3 individuals per bin. The identified germline analysis consisted of patients who consented to secondary germline analysis. For all patients, pathology records were reviewed and confirmed at MSKCC. Demographic and clinical data were manually extracted from electronic medical records by 2 clinical research coordinators (Y.T. and K.C.); data were extracted into standard fields in an Excel spreadsheet (designed by G.Y.K. and Y.K.). There was no a priori study hypothesis. Any ambiguous clinical data were adjudicated (G.Y.K.). Race/ethnicity was based on self-reporting by patients and was extracted because of potential differences in germline variants based on race/ethnicity.

### Germline Analysis

Normal nontumor DNA from blood was analyzed using a 76- or 88-gene panel (eAppendix in the Supplement), including all cancer-predisposing genes identified by the American College of Medical Genetics and Genomics (ACMG) guidelines.^5^ DNA was sequenced and variants reported as described previously.^6^ Variant rediscovery was performed using standard germline variant-calling methods with stringent quality controls. Variant interpretation relied on an initial pass by the automated variant classification algorithm PathoMAN^7^ and subsequent manual curation (Y.K.) based on ACMG criteria.^8^ Germline variants were classified as having high (relative risk, >4), moderate (relative risk, 2–4), or low (relative risk, <2) penetrance and/or as being recessive or of uncertain clinical actionability based on known associated risks as previously published (eAppendix in the Supplement). For the identified analysis, patients with LP/P variants were offered genetic counseling through the Clinical Genetics Service. In patients for whom LP/P variants were identified, the tumor was subsequently assessed for somatic variants and/or loss of heterozygosity in the corresponding gene or genes.

### Statistical Analysis

All patients with an esophageal squamous cell carcinoma or an esophagogastric adenocarcinoma who had MSK-IMPACT testing performed were included in this analysis. Baseline clinicopathologic characteristics were summarized separately within the identified and anonymized cohorts using median (range) for continuous variables and number (percentage) for categorical features. The χ^2^ test was used to assess differences in overall germline variant prevalence (positive vs negative) as well as the prevalence of high- or moderate-penetrance genetic variants according to tumor location (esophageal vs gastric) and age at diagnosis (≤50 vs >50 years), and exact binomial 95% CIs around the estimated proportions were provided. The Cochran-Mantel-Haenszel test was used to study associations between age at diagnosis (≤50 vs >50 years) and overall germline alteration while stratifying by tumor location. For categorical variables with any expected frequency of less than 5, the Fisher exact test was used.

Overall survival (OS) and progression-free survival (PFS) were analyzed among 304 identified patients who were treated with first-line platinum therapy. Both OS and PFS were calculated from the date of first-line platinum therapy initiation until date of death for OS or first progression or death, whichever occurred first, for PFS and compared between subgroups using the log-rank test. The end date for follow-up was July 31, 2020.

All statistical analyses were conducted using R, version 3.6.0 (R Foundation for Statistical Computing). *P* values were calculated using a 2-sided test with a cutoff of *P* < .05 to indicate statistical significance.

## Results

### Patient and Disease Characteristics

A total of 515 patients (median age, 59 years; range, 18-87 years; 368 [71.5%] male; 398 [77.3%] White) were identified. Patient demographic characteristics are given in the Table. Tumors in 501 patients (97.3%) were adenocarcinomas. A total of 344 patients (66.8%) had metastatic disease at the time of diagnosis. Initial therapy consisted of a platinum-based regimen in 478 patients (92.8%).

**Table.** Demographic and Clinicopathologic Characteristics of the Study Patients^a^

Characteristic	All patients (N = 515)	Anonymized (n = 189)	Identified (n = 326)
Age at diagnosis, median (range), y	59 (18-87)	59 (23-87)	58 (18-85)
Sex			
Male	368 (71.5)	139 (73.5)	229 (70.2)
Female	147 (28.5)	50 (26.5)	97 (29.8)
Race/ethnicity			
White	398 (77.3)	145 (76.7)	253 (77.6)
Black, Hispanic, or unknown	74 (14.4)	26 (13.8)	48 (14.7)
Asian	43 (8.3)	18 (9.5)	25 (7.7)
Ashkenazi Jewish			
Yes	53 (10.3)	6 (3.2)	47 (14.4)
No	370 (71.8)	143 (75.7)	227 (69.6)
Unknown	92 (17.9)	40 (21.2)	52 (16.0)
Primary site			
Esophageal	161 (31.3)	95 (50.3)	66 (20.2)
Gastroesophageal junction	111 (21.6)	9 (4.8)	102 (31.3)
Gastric	243 (47.2)	85 (45.0)	158 (48.5)
Histologic subtype			
Adenocarcinoma	501 (97.3)	189 (100)	312 (95.7)
Squamous cell carcinoma	14 (2.7)	0	14 (4.3)
Stage at diagnosis			
Locally advanced	171 (33.2)	52 (27.5)	119 (36.5)
Metastatic	344 (66.8)	137 (72.5)	207 (63.5)
Microsatellite instability status			
Unstable	33 (6.4)	5 (2.6)	28 (8.6)
Stable	472 (91.6)	184 (97.4)	288 (88.3)
Unknown	10 (1.9)	0	10 (3.1)
Initial chemotherapy			
Platinum based	478 (92.8)	172 (91.0)	306 (93.9)
Non–platinum based	34 (6.6)	17 (9.0)	17 (5.2)
None or non–chemotherapy based	3 (0.6)	0	3 (0.9)

^a^ Data are presented as number (percentage) of patients unless otherwise indicated.

### Germline Variant Prevalence

**Figure 1.**
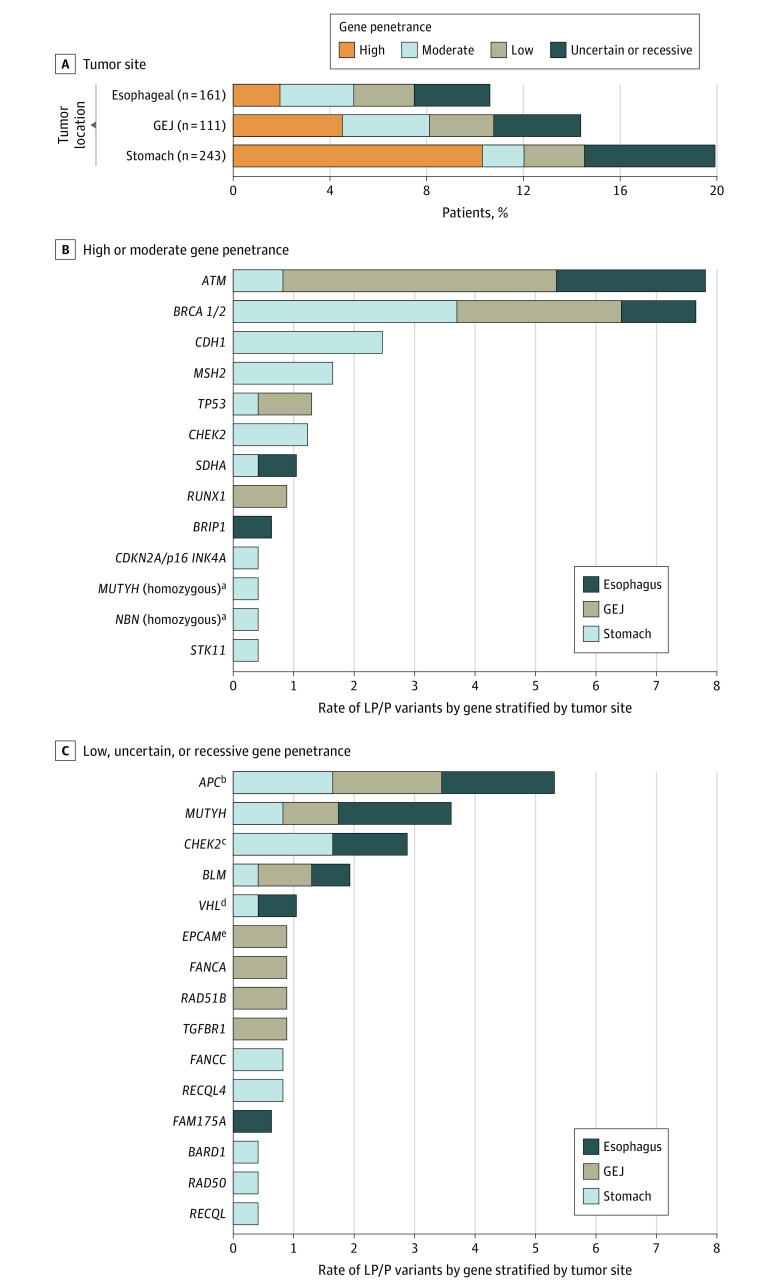
Prevalence of Germline Alterations by Percentage GEJ indicates gastroesophageal junction; LP/P, likely pathogenic or pathogenic. ^a^Present as homozygous variants: *NBN* c.481-2A>T and recurrent *MUTYH* germline variant c.536A>G (p.Tyr179Cys), respectively. ^b^*APC* c.3920T>A (p.Ile1307Lys), common founder variant among the Ashkenazi Jewish population, considered to be of low penetrance. ^c^*CHEK2* c.470T>C (p.Ile157Thr), considered to be of uncertain clinical actionability. ^d^*VHL* c.598C>T (p.Arg200Trp) germline variant associated with recessive Chuvash type polycythemia and not with von Hippel Lindau syndrome and therefore not categorized as high penetrance. ^e^*EPCAM* c. 325C>T (p.Gln109*) germline variants not reported in the literature; *EPCAM* loss-of-function variants (other than 3′*EPCAM* deletions associated with Lynch syndrome) have been associated with autosomal recessive congenital tufting enteropathy.

Among the 515 patients, the tumor was located in the esophagus in 161 patients (31.3%), the GEJ in 111 patients (21.6%), and the stomach in 243 patients (47.2%) (Table). We observed a borderline association between the presence of LP/P germline variants and tumor location (17 [10.6%]; 95% CI, 6.3%-15.4% in esophageal cancer; 16 [14.4%]; 95% CI, 8.5%-22.3% in GEJ cancer; and 48 [19.8%]; 95% CI, 14.9%-25.3% in gastric cancer; *P* = .04) (Figure 1A). Because GEJ tumors are likely to reflect a mix of true GEJ tumors as well as proximal gastric and distal esophageal cancers and can be impossible to clinically distinguish with certainty, we limited our statistical comparison of variant prevalences to esophageal vs gastric cancer; patients with gastric cancers (48 [19.8%]; 95% CI, 14.9%-25.3%) were more likely to carry germline variants compared with patients with esophageal cancer (17 [10.6%]; 95% CI, 6.3%-15.4%; *P* = .02).

We next grouped genetic variants into high, moderate, low, or uncertain penetrance or recessive cancer susceptibility genes and compared their distributions among tumor locations (Figure 1A). In subgroup analyses, LP/P variants in high- and moderate-penetrance genes were significantly more prevalent in patients with gastric cancer (29 [11.9%]; 95% CI, 8.1%-16.7%) vs patients with esophageal cancer (8 [5.0%]; 95% CI, 2.2%-9.6%; *P* = .03). Moreover, high-penetrance germline LP/P variants were present in 25 patients with gastric cancer (10.3%; 95% CI, 6.8%-14.8%) but only 3 patients with esophageal cancer (1.9%; 95% CI, 0.38%-5.3%; *P* = .001) and 5 patients with GEJ cancer (4.5%; 95% CI, 1.5%-10.2%). The frequency of LP/P variants in moderate-penetrance (4 [1.6%] in gastric cancer, 4 [3.6%] in GEJ cancer, and 5 [3.1%] in esophageal cancer) and low-penetrance (6 [2.5%] in gastric cancer, 3 [2.7%] in GEJ cancer, and 4 [2.5%] in esophageal cancer) genes, as well as monoallelic autosomal recessive (designating carrier status) and uncertain genetic variants (13 [5.4%] in gastric cancer, 4 [3.6%] in GEJ cancer, and 5 [3.1%] in esophageal cancer for combined recessive and uncertain variants), was not statistically different across the 3 tumor locations, in line with the presumption that most of these variants were incidental and did not contribute to cancer development in these patients.

We also assessed germline variant prevalence in patients with early-onset cancer, namely in patients diagnosed with esophagogastric cancer at 50 years or younger, a cutoff selected based on Surveillance Epidemiology and End Results data indicating that the age of 50 years is more than 1 SD below the mean age at esophagogastric cancer diagnosis.^9^ In the overall cohort, 29 patients 50 years or younger (21.0%; 95% CI, 14.5%-28.8%) vs 52 patients older than 50 years (13.8%; 95% CI, 10.5%-17.7%) harbored a germline alteration (*P* = .046). After stratifying by cancer location, although a higher alteration prevalence in those with early-onset disease was present in all 3 groups (19 [22.4%] in gastric cancer, 4 [17.4%] in GEJ cancer, and 6 [20.0%] in esophageal cancer in patients diagnosed at 50 years or younger vs 28 [17.8%] in gastric cancer, 13 [14.6%] in GEJ cancer, and 11 [8.4%] in esophageal cancer in patients with late-onset cancers), this was no longer statistically significant, possibly as a result of low numbers of patients diagnosed at 50 years or younger in each subtype (*P* = .11).

Given the enrichment of patients of Ashkenazi Jewish descent within the MSKCC patient population, we also assessed alteration status with respect to ancestry in the 326 patients who underwent identified germline analysis, of whom 47 (14.4%) self-identified as Ashkenazi Jewish (Table). The germline variant prevalence found among 227 self-identified non–Ashkenazi Jewish patients (38 [16.7%]) was comparable to the prevalence in the overall cohort (81 [15.7%]).

Figure 1B and C show the spectrum of germline LP/P variants observed in our cohort according to penetrance. The LP/P variants were observed in 15 high- or moderate-penetrance genes, with *ATM* (OMIM 607585) (n = 11), *BRCA1* (OMIM 113705) (n = 7), *BRCA2* (OMIM 600185) (n = 7), *CDH1* (OMIM 192090) (n = 6), and *MSH2* (OMIM 609309) (n = 4) being the most frequently altered genes. *ATM* was the most commonly altered single gene, with an alteration prevalence of 2.1% in the entire cohort. *ATM* LP/P variants occurred in 6 patients (4.3%; 95% CI, 1.6%-9.1%) with early-onset esophagogastric cancer vs 5 (1.3%; 95% CI, 0.4%-3.1%; *P* = .08) of those with late-onset esophagogastric cancer.

### Inheritance of Variants in Frequently Altered Genes

For 42 of 55 patients (76.4%) in whom germline alterations were identified, clinical genetic testing based on National Comprehensive Cancer Network and ACMG guidelines^4,10^ would have missed germline variants, including 16 of 29 (55.2%) of the high- and moderate-penetrance variants.

**Figure 2.**
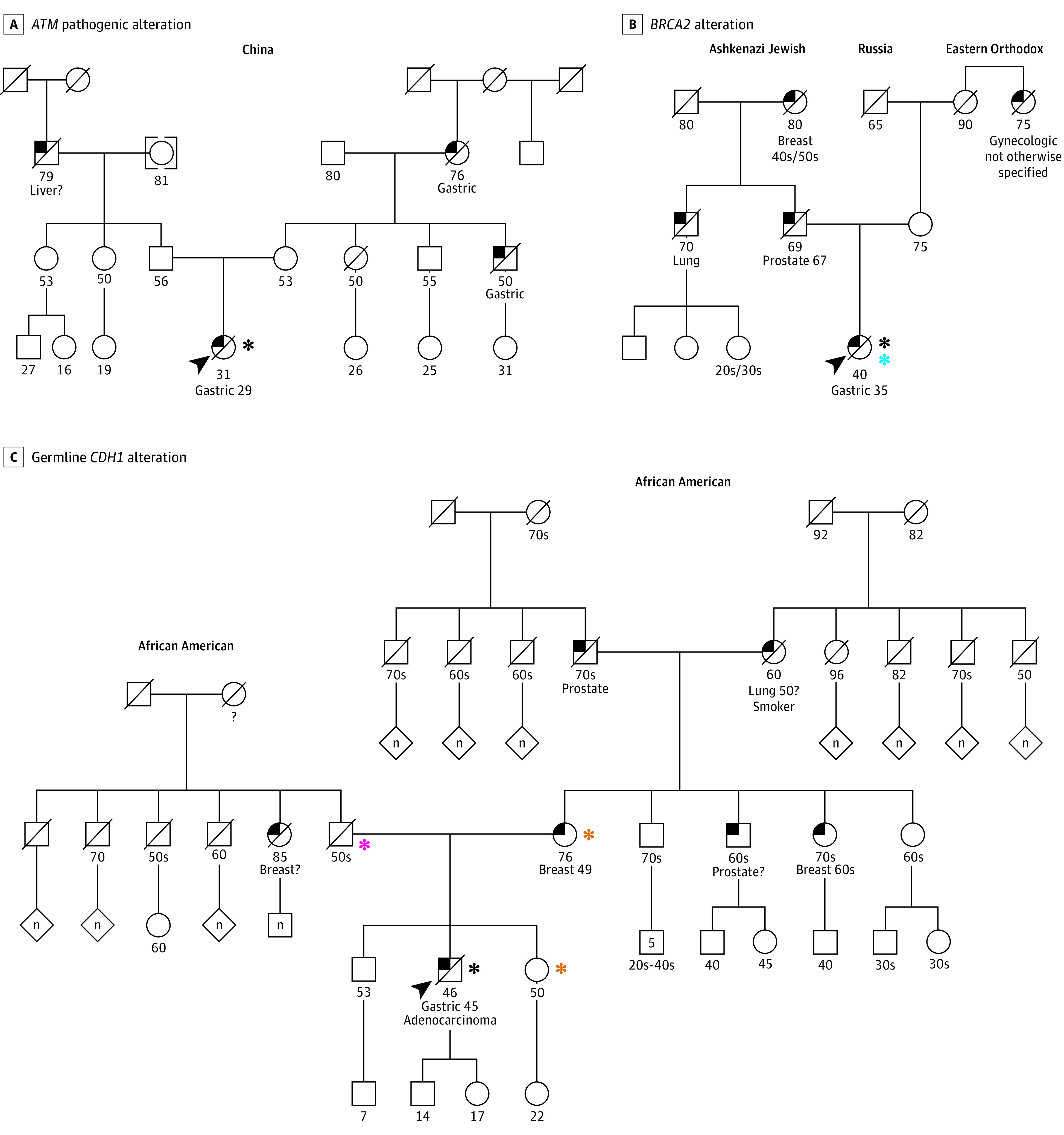
Pedigrees of Selected Patients With Germline Alterations A, *ATM* pathogenic alteration in a 29-year-old woman with stage IV gastric signet ring cell adenocarcinoma treated with folinic acid, fluorouracil, and oxaliplatin (FOLFOX) with treatment discontinued after 6 months because of toxic effects. Overall survival was 9.5 months. The identification of an *ATM* pathogenic alteration represented an incidental finding in a relative with familial gastric cancer. B, *BRCA2* alteration in a 35-year-old woman with stage IV gastric cardia cancer to the lymph nodes with complete response to epirubicin, oxaliplatin, and capecitabine chemotherapy that lasted for 16 months. Overall survival was 53.5 months. In addition to the germline *BRCA2* alteration, a somatic *BRCA2* alteration was also identified, presumed to be the second hit in the context of a cancer caused by the germline mutation. C, Germline *CDH1* alteration in a 45-year-old man with stage IV gastric signet ring cell carcinoma treated with FOLFOX for 9 months before progression. He remained alive 10.4 months into treatment. A germline *CDH1* alteration, presumably inherited from the paternal lineage without familial gastric cancer, was identified. Because he did not meet genetic testing criteria for *CDH1*, it is considered an incidental finding. Square indicates male; circle, female; bracket around a circle, adopted individual; slash, deceased; black upper left portion of a square or circle, cancer-affected individual; arrowhead, a family member who underwent matched tumor and germline sequencing; black asterisk, germline-positive status; blue asterisk, presence of a second somatic alteration; pink asterisk, obligate carrier; and gold asterisk, confirmed germline-negative status.

Six patients 50 years or younger had *ATM* LP/P variants (2 with gastric cancer, 1 with GEJ cancer, and 3 with esophageal cancer), of whom family gastric cancer history was positive in all 3 patients with available histories (2 with gastric cancer and 1 with GEJ cancer). Figure 2A depicts the pedigree of a family with an *ATM* alteration and history of gastric cancer, with the proband diagnosed with gastric cancer at 29 years of age.

The frequencies of variants in *BRCA1* and *BRCA2* were each 1.4%. A total of 12 *BRCA1/2* variants (85.7%) were found in patients with gastric and GEJ cancers. Of the 14 *BRCA1/BRCA2* LP/P variant carriers, 8 had family cancer histories available and 7 met criteria for hereditary breast and ovarian cancer syndrome.^11^ Only 4 (28.6%) were patients diagnosed with early-onset disease at 50 years or younger. Figure 2B is a representative pedigree from a patient with very early-onset gastric cancer and a *BRCA2* alteration. Tumor sequencing revealed a somatic alteration in *BRCA2*, a presumed second hit, suggesting that the germline event was likely a driver of tumorigenesis.

LP/P variants in *CDH1* were identified in 6 patients with gastric cancer. Figure 2C shows the pedigree of a patient with gastric signet ring cell adenocarcinoma who did not meet clinical criteria for *CDH1* germline analysis but nonetheless harbored a pathogenic *CDH1* alteration, presumably inherited from the paternal lineage, without a family history suggestive of hereditary diffuse gastric cancer.

Given potential clinical implications for treatment response, we also evaluated the prevalence of LP/P germline variants in genes associated with DNA damage repair (DDR) and found that 27 patients overall (5.2%) and 16 patients (6.6%) with gastric cancer, 5 (4.5%) with GEJ cancer, and 6 (3.7%) with esophageal cancer harbored variants in these genes.

### Association With Treatment Outcomes

We evaluated whether patients with variants in homologous recombination deficiency (HRD) and DDR genes had better outcomes when treated with platinum-based chemotherapy, the cornerstone of systemic treatment for locally advanced and metastatic esophagogastric cancer (eFigure in the Supplement). We found no difference in OS or PFS at 12 months between patients with germline alterations in HRD (OS, 68%; 95% CI, 46%-100%; PFS, 43%; 95% CI, 23%-78%) vs DDR (OS, 56%; 95% CI, 37%-87%; PFS, 35%; 95% CI, 19%-67%) vs other genes (OS, 86%; 95% CI, 72%-100%; PFS, 62%; 95% CI, 44%-87%) vs no germline variants (OS, 61%; 95% CI, 55%-68%; PFS, 49%; 95% CI, 43%-55%).

We also compared PFS and OS between patients with or without *BRCA1/2* germline alterations who received platinum-based treatment and found no difference. However, our study may have been underpowered to detect such differences.

## Discussion

This cross-sectional study found that pathogenic germline variants were significantly more common in patients with gastric compared with esophageal cancer, with carcinogenesis in the latter more closely linked to exogenous or environmental risk factors. Specifically, the study found not only a higher prevalence of actionable germline variants but also a 5-fold greater prevalence of high-penetrance variants in patients with gastric cancer compared with esophageal cancer (10% vs 2%).

The prevalence of germline LP/P variants in GEJ tumors was intermediate between those in gastric and esophageal cancers. The Cancer Genome Atlas analysis of esophageal cancers found a gradual transition of molecular subtypes from the distal esophagus through the GEJ into the proximal stomach.^12^ In addition, GEJ cancers are heterogeneous because ascertaining the true epicenter of a tumor at the esophagogastric junction can be impossible, even in patients who undergo endoscopy.

Interestingly, a variant in the moderate-penetrance *ATM* gene was identified in approximately 2% of the overall cohort. Although germline variants in *ATM* are associated with increased risk of breast and pancreatic cancer,^13,14,15^ their association with esophagogastric cancer is less certain. Huang et al^16^ found that 2.7% of patients with gastric cancer sequenced via The Cancer Genome Atlas harbored an *ATM* variant, similar to the findings of the current study and significantly higher than in control populations. *ATM* variant prevalence in esophagogastric cancer appears to be similar to its prevalence in unselected patients with pancreatic cancer (2.3%).^15^ As highlighted by the pedigree (Figure 2A), these variants may occur in the setting of familial gastric cancer.

This study also identified 7 *BRCA1* and 7 *BRCA2* germline variants. Most (85.7%) of the *BRCA1/2* carriers had gastric or GEJ tumors, in line with prior studies^17,18,19,20^ that suggested the potential role of *BRCA* in gastric cancer susceptibility. Although most *BRCA1/2* carriers developed later-onset esophagogastric cancer, 28.6% of patients had early-onset disease, including a 35-year-old *BRCA2* carrier whose tumor also carried a second somatic *BRCA2* variant, suggesting that the germline *BRCA2* variant contributed to carcinogenesis. Given the US Food and Drug Administration approval of poly(adenosine diphosphate) ribose polymerase (PARP) inhibitors in advanced *BRCA*-associated ovarian, breast, prostate, and pancreatic cancers,^21,22,23,24^ the identification of germline variants in *BRCA1/2* may have future implications for treatment by being the basis for enrolling such patients in therapeutic studies of PARP inhibitors.

A total of 76.4% of patients did not have a personal and/or a family history of cancer that would have warranted clinical genetic testing based on current National Comprehensive Cancer Network and ACMG guidelines, including 55.2% of patients who harbored variants in high- or moderate-penetrance genes. Given the inadequate sensitivity of existing clinical guidelines, as well as barriers to access to genetic evaluation, this study suggests that any patient with gastric cancer in the US who undergoes somatic tumor genetic testing should also undergo germline testing. The identification of a clinically actionable variant would have clear screening implications for any at-risk family members. In contrast, the prevalence of a high-penetrance germline variant in the esophageal cancer cohort was less than 2%, which, on the basis of these data, does not justify routine testing in such patients. Because the prevalence of high-penetrance variants was between these two values in the GEJ population (4.5%) and given the challenge in accurately classifying such tumors as belonging exclusively to the esophagus or the stomach, germline testing for patients with GEJ tumors can also be considered.

The survival analysis of patients with germline variants in HRD and DDR pathways and in particular the *BRCA1/2* genes did not support enhanced responsiveness to treatment with platinum-containing chemotherapy regimens, whose mechanism of action involves damaging DNA. This finding contrasts with studies in pancreatic cancers^25^ and likely results from lack of power to detect a survival difference; only 5 patients were identified with a *BRCA1/2* variant with metastatic esophagogastric adenocarcinoma who received first-line platinum-based therapy (other patients were anonymized or had squamous cell carcinoma tumors or locally advanced disease). Their median disease-free survival was 13.7 months (range, 5.8-16.0 months), which suggests a more favorable outcome than for chemotherapy because the median disease-free survival while receiving first-line chemotherapy is normally 4 to 6 months.^2^ In addition, it is possible that not all germline *BRCA* variants identified contributed to the development of these cancers and that the grouping of esophageal, GEJ, and gastric tumors together obscures the potential differential effect of *BRCA* variants by tumor location. Because this study could not assess for somatic *BRCA* variants or loss of heterozygosity in all cases, the role of the germline *BRCA* variant in each patient could not be definitively established.

### Limitations

This study has limitations, including its retrospective nature, a median age of diagnosis 10 years younger than the general US population, and a higher-than-average representation of patients with metastatic disease at the time of diagnosis (approximately two-thirds vs half).^2^ This high prevalence is related to the fact that next-generation sequencing somatic and germline alteration testing have, up to now, been preferentially performed in patients with metastatic disease, in part to identify actionable alterations for targeted therapies. The patient population treated at MSKCC in the New York area also includes higher-than-average proportions of patients with Ashkenazi Jewish ancestry or Asian ethnicity, so these results may not be applicable to other patient populations. In addition, *CTNNA1* (OMIM 116805), a recently implicated gene associated with inherited diffuse gastric cancer, was not included on our germline panel, possibly leading to underestimation of overall germline variant prevalence.

## Conclusions

This large retrospective analysis suggests that pathogenic germline variants may be significantly more common in gastric than in esophageal cancer, with GEJ tumors having an intermediate prevalence, as well as in patients with esophagogastric cancer diagnosed at 50 years or younger, although this observation was of borderline statistical significance and needs to be validated. Because many of the cancer predisposition syndromes identified have significant implications for cancer surveillance and risk reduction in at-risk relatives and could influence therapy selection in patients affected by cancer, germline testing should be considered in patients with GEJ or gastric cancer and any patient with esophagogastric cancer diagnosed at 50 years or younger. Identifying germline HRD and DDR pathway alterations also permits prioritization of standard platinum-based therapy and experimental PARP inhibitors for these patients. Finally, a large number of LP/P variants in *ATM* were identified in this cohort, especially in patients with early-onset disease, and this observation warrants confirmation in other data sets.

## Notes

Supplement. **eAppendix.** List of Genes in MSK-IMPACT 76 Gene Panel **eFigure.** Survival Following Platinum-Based Chemotherapy According to Alteration Class
