# Supplementary material for: Prevalence of Germline Alterations on Targeted Tumor-Normal Sequencing of Esophagogastric Cancer
Source: JAMA Netw Open. 2021 Jul 12;4(7):e2114753. doi: 10.1001/jamanetworkopen.2021.14753 (PMC8276088; doi:10.1001/jamanetworkopen.2021.14753)
Supplement: Supplement. — eAppendix. List of Genes in MSK-IMPACT 76 Gene Panel eFigure. Survival Following Platinum-Based Chemotherapy According to Alteration Class [file jamanetwopen-e2114753-s001.pdf]

Supplementary Online Content

Ku GY, Kemel Y, Maron SB, et al. Prevalence of germline alterations on targeted tumor-normal sequencing of esophagogastric cancer. *JAMA Netw Open*. 2021;4(7):e2114753. doi:10.1001/jamanetworkopen.2021.14753

eAppendix. List of Genes in MSK-IMPACT 76 Gene Panel

eFigure. Survival Following Platinum-Based Chemotherapy According to Alteration Class

This supplementary material has been provided by the authors to give readers additional information about their work.

© 2021 Ku GY et al. *JAMA Network Open.*

eAppendix. List of Genes in MSK-IMPACT 76 Gene Panel

	**Genes_76**
1	ALK
2	APC
3	ATM
4	BAP1
5	BARD1
6	BLM
7	BMPR1A
8	BRCA1
9	BRCA2
10	BRIP1
11	CDH1
12	CDK4
13	CDKN2A
14	CHEK2
15	DICER1
16	EGFR
17	EPCAM
18	FAM175A (Abraxas)
19	FH
20	FLCN
21	GATA2
22	GREM1
23	HRAS
24	JAK2
25	KIT
26	KRAS
27	MAX
28	MEN1
29	MET
30	MITF
31	MLH1
32	MRE11A
33	MSH2
34	MSH6
35	MUTYH
36	NBN
37	NF1
38	NF2
39	NRAS

© 2021 Ku GY et al. *JAMA Network Open.*

	Genes_76
40	PALB2
41	PAX5
42	PDGFRA
43	PHOX2B
44	PMS2
45	POLE
46	PTCH1
47	PTEN
48	RAD50
49	RAD51
50	RAD51B
51	RAD51C
52	RAD51D
53	RB1
54	RECQL4
55	RET
56	RUNX1
57	SDHA
58	SDHAF2
59	SDHB
60	SDHC
61	SDHD
62	SMAD3
63	SMAD4
64	SMARCA4
65	SMARCB1
66	STK11
67	SUFU
68	TERT
69	TGFBR1
70	TGFBR2
71	TMEM127
72	TP53
73	TSC1
74	TSC2
75	VHL
76	WT1

© 2021 Ku GY et al. *JAMA Network Open.*

List of genes in MSK-IMPACT 88 gene panel

	Genes_88
1	ALK
2	APC
3	ATM
4	BAP1
5	BARD1
6	BLM
7	BMPR1A
8	BRCA1
9	BRCA2
10	BRIP1
11	CDH1
12	CDC73
13	CDK4
14	CDKN2A
15	CEBPA
16	–CHEK2
17	DICER1
18	EGFR
19	EPCAM
20	ERBB2
21	ERCC3
22	ETV6
23	FAM175A (Abraxas)
24	FANCA
25	FANCC
26	FH
27	FLCN
28	GATA2
29	HOXB13
30	HRAS
31	KIT
32	KRAS
33	MAX
34	MEN1
35	MET
36	MITF
37	MLH1
38	MRE11A
39	MSH2

© 2021 Ku GY et al. *JAMA Network Open.*

	Genes_88
40	MSH3
41	MSH6
42	MUTYH
43	NBN
44	NF1
45	NF2
46	NRAS
47	NTHL1
48	PALB2
49	PAX5
50	PDGFRA
51	PHOX2B
52	PMS2
53	POLD1
54	POLE
55	PTCH1
56	PTEN
57	RAD50
58	RAD51
59	RAD51B
60	RAD51C
61	RAD51D
62	RB1
63	RECQL
64	RECQL4
65	RET
66	RTEL1
67	RUNX1
68	SDHA
69	SDHAF2
70	SDHB
71	SDHC
72	SDHD
73	SMAD3
74	SMAD4
75	SMARCA4
76	SMARCB1
77	STK11
78	SUFU
79	TERT

© 2021 Ku GY et al. *JAMA Network Open.*

	Genes_88
80	TGFBR1
81	TGFBR2
82	TMEM127
83	TP53
84	TSC1
85	TSC2
86	VHL
87	WT1
88	YAP1

© 2021 Ku GY et al. *JAMA Network Open.*

eFigure. Survival Following Platinum-Based Chemotherapy According to Alteration Class

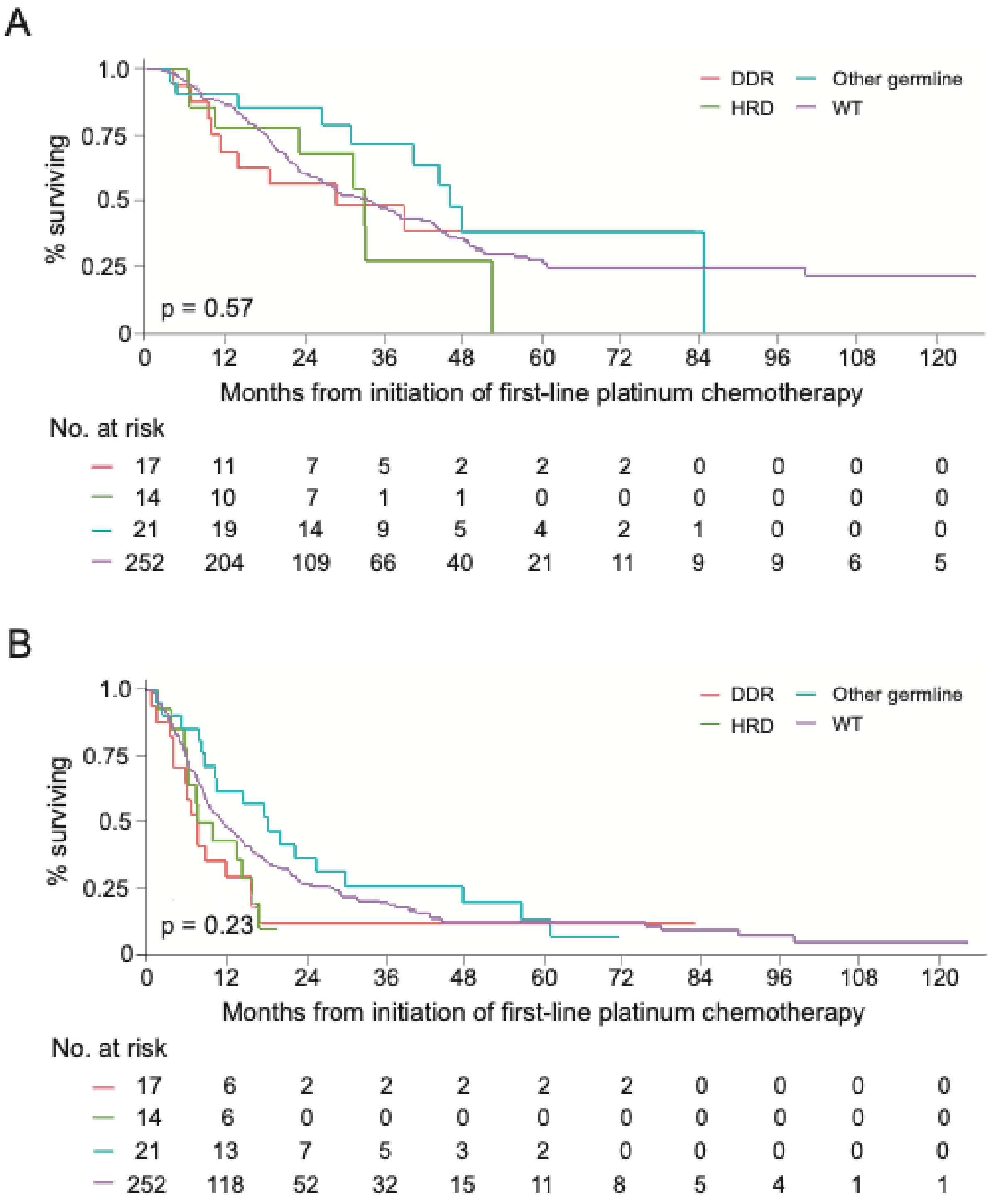

© 2021 Ku GY et al. *JAMA Network Open.*
